# Three-Dimensional Cartilage Tissue Engineering Using Placenta-Derived Extra-Embryonic Mesenchymal Stem Cells: From Isolation to Differentiation

**DOI:** 10.3390/biomedicines13092291

**Published:** 2025-09-18

**Authors:** Cem Mujde, Atil Bisgin

**Affiliations:** Department of Biotechnology, Institute of Natural and Applied Sciences, Cukurova University, Adana 01330, Türkiye; cemmujde@gmail.com

**Keywords:** extra-embryonic mesenchymal stem cells, 3D cell culture, artificial tissue engineering, regenerative medicine, biomaterials

## Abstract

**Background/Objectives**: Mesenchymal stem cells (MSCs) offer promising prospects for novel treatment modalities in cellular therapies and artificial organ production. Despite a surge in artificial tissue research, there is a dearth of comprehensive studies detailing the entire process from stem cells to tissue production, coupled with a scarcity. This study, however, presents the utility of extra-embryonic MSCs derived from placental tissue, traditionally considered as medical waste. **Methods**: Within a 3-dimensional cell culture system, histological assessments, and comprehensive optimization studies, the entire process required for artificial tissue production is addressed. **Results**: The results obtained are encouraging regarding the advancement of cellular therapies and artificial tissue engineering. However, challenges such as biopolymer degradation highlight the necessity for multistep approaches. Each analysis within this study delves into the discussion and optimization of key steps in artificial tissue production. **Conclusions:** Consequently, this study not only represents one of the first of its kind but also lays the groundwork for future investigations into relevant clinical applications.

## 1. Introduction

Mesenchymal stem cells (MSCs) have garnered considerable attention as a promising source for cell therapies in clinical applications. This is due to their remarkable abilities to self-renew and differentiate into various specialized cell types, making them invaluable for a wide range of therapeutic purposes, including tissue repair and regeneration [1,2]. MSCs are widely distributed in numerous tissues, including the umbilical cord [3], bone marrow [4], cartilage [5], adipose tissue [6], placenta [7], and dental pulp [8], among others. Among these sources, the placenta stands out as an especially valuable reservoir of MSCs. It offers a non-invasive method of collection and presents minimal ethical concerns, as it is typically categorized as postpartum medical waste. Placenta-derived extra-embryonic MSCs have demonstrated superior proliferative capacity, extended longevity, and enhanced differentiation potential compared to MSCs derived from other tissues, such as bone marrow [9,10]. These attributes make placenta-derived MSCs an ideal candidate for various therapeutic applications, particularly in regenerative medicine.

The defining characteristics of MSCs include their multipotency, meaning their ability to differentiate into several cell types, such as osteocytes, chondrocytes, and adipocytes, as well as their capacity to adhere to plastic surfaces and express specific cell surface markers [11,12]. Placenta-derived extra-embryonic MSCs, in particular, exhibit features that make them highly suitable for clinical applications. These include their ability to self-renew, their plasticity, their ease of isolation, and minimal ethical concerns [10,11]. These cells hold great promise for developing novel therapeutic strategies, especially in the field of tissue engineering.

In the context of cartilage regeneration, chondrocytes play a pivotal role in maintaining the structure and function of cartilage tissues within joints. These cells are responsible for the synthesis of collagen and the extracellular matrix (ECM), both of which are critical for cartilage integrity. Mesenchymal stem cells differentiate into prechondrocytes, which then develop into chondroblasts. Chondroblasts secrete collagenous fibrils and other components of the ECM, such as proteoglycans, which are essential for maintaining the structure and mechanical properties of cartilage [12].

The ECM of mature articular cartilage is directly linked to the volume and function of chondrocytes. It is composed of three primary types of macromolecules: collagen and elastin fibers, proteoglycans, and glycoproteins. Collagen, particularly type II collagen, is the dominant fiber found in the ECM of hyaline cartilage, comprising more than 90% of the collagen present [13]. The ECM also contains non-collagenous elements such as glycosaminoglycans (GAGs), which include chondroitin sulfate, keratan sulfate, dermatan sulfate, heparan sulfate, and hyaluronan (or hyaluronic acid). These molecules are negatively charged, which helps them repel each other while attracting water and ions (e.g., Ca^2+^ and Na^+^), thus contributing to the hydration and mechanical properties of the ECM [14].

Tissue engineering has experienced rapid advancements in recent years, especially in the field of cartilage regeneration, with a focus on both human and animal models [15,16]. Numerous studies have explored the combination of different cell sources, biocompatible scaffolds, growth factors, and novel techniques to create cartilage tissue ex vivo for subsequent implantation in vivo [17,18]. Biodegradable polymers, such as poly(lactic-*co*-glycolic acid) (PLGA), have been identified as effective scaffolds for 3D cell culture, providing a conducive environment for chondrogenic differentiation and ECM production [19]. In contrast, chondrocytes that are expanded in two-dimensional (2D) cultures often undergo dedifferentiation, leading to a reduction in cartilage matrix synthesis and loss of their cartilage-specific phenotype [20].

Growth factors play a critical role in directing MSC differentiation into specific phenotypes, especially in cartilage metabolism. Chondrogenesis, the process of cartilage formation, is regulated by several growth factors, including members of the transforming growth factor-beta (TGF-β) superfamily. This family comprises over 30 members that are involved in cell growth, differentiation, migration, and apoptosis. TGF-β1, in particular, has been extensively studied for its role in chondrogenesis and cartilage ECM synthesis [21]. In 3D culture systems, TGF-β1 has been shown to enhance the differentiation of MSCs into chondrocytes, promoting the synthesis of key ECM components such as collagen and glycosaminoglycans (GAGs) in both human and animal models [22,23,24,25].

This study aims to explore the potential of placenta-derived MSCs in generating artificial cartilage tissue within a 3D cell culture system. Biopolymers, which are biodegradable and biocompatible, play a crucial role in tissue engineering by providing scaffolds that support cell attachment and tissue formation. Commonly used biopolymers such as poly(glycolic acid) (PGA), polylactic acid (PLA), poly(lactic-*co*-glycolic acid) (PLGA), and polycaprolactone (PCL) have proven to be effective substrates for the cultivation of cells in a 3D environment, especially when combined with 3D printing technologies [26,27,28].

The term “artificial tissue” refers to a 3D assembly of cells that is designed to mimic the structure and function of native tissues [29,30]. These tissues or organoids are typically derived from stem cells, including embryonic stem cells (ESCs), induced pluripotent stem cells (iPSCs), and tissue-specific adult stem cells, such as MSCs and adipose tissue-derived stem cells (ASCs) [31]. Recent breakthroughs in tissue engineering have led to the increasing use of artificial tissues in disease modeling, cancer research, and the study of human development [32].

The transition from traditional 2D cell cultures to more advanced 3D cell cultures represents a significant step forward in the field of tissue engineering [33]. Compared to 2D cultures, 3D cell culture systems more accurately mimic the in vivo environment, offering enhanced morphological features and better representation of cellular behaviors such as differentiation, proliferation, viability, and responses to external stimuli [34,35]. These systems provide a more realistic platform for evaluating tissue functions and drug responses.

While traditional bioreactors are used for large-scale production of biological materials, dynamic bioreactors have emerged as an essential tool in 3D cell culture. These bioreactors can regulate environmental conditions such as pH, gas concentrations, nutrient levels, and shear forces, enabling the optimization of cell culture processes in a more controlled and efficient manner [36,37,38]. Rotary cell culture systems (RCCSs), such as the Slow-Turning Lateral Vessel (STLV), have proven to be particularly effective in supporting 3D cell cultures by facilitating nutrient diffusion and promoting ECM synthesis [39,40,41].

In light of these advancements, our study seeks to harness the potential of placenta-derived extra-embryonic MSCs in a 3D cell culture system for the creation of artificial cartilage tissue. By combining these MSCs with biocompatible scaffolds and growth factors, we aim to contribute to the growing field of tissue engineering and regenerative medicine, paving the way for novel treatments in cartilage repair and regeneration.

## 2. Materials and Methods

### 2.1. Isolation and Culture of Human Placenta-Derived MSCs

Placental tissues were obtained from healthy pregnant women who underwent elective cesarean sections at Cukurova University Balcali Hospital. All participants were confirmed to have seronegative status and uncomplicated pregnancies. The study protocol was approved by the Cukurova University Institutional Ethics Committee (Approval No: 80/31 August 2018), in compliance with the Declaration of Helsinki. Informed consent was obtained from all participants prior to their inclusion in the study.

**Figure 1:**
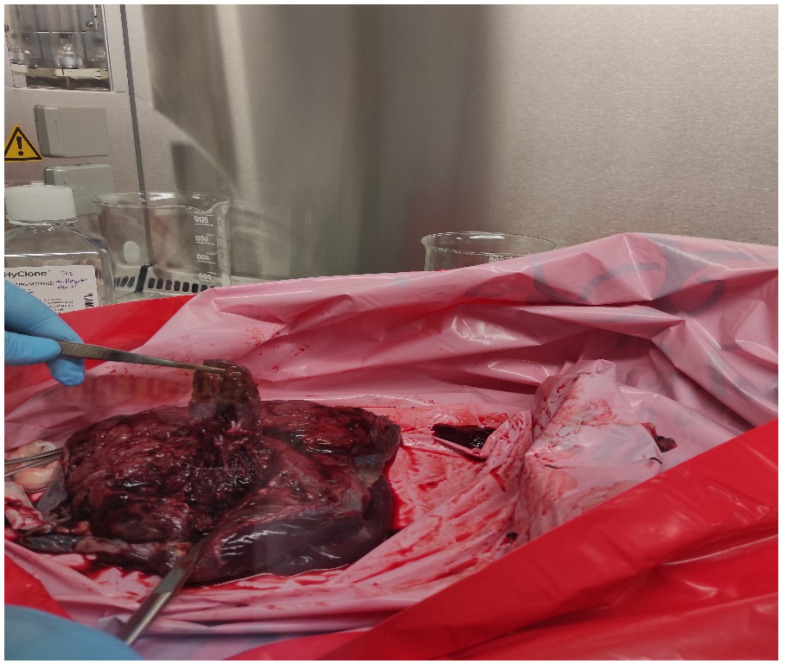
Chorionic villus dissections from the placenta in the class II safety cabin.

Under aseptic surgical conditions, the placenta was collected and transported on ice (4 °C) to the Tissue & Cell Culture Laboratory at Cukurova University AGENTEM for further processing. The isolation process followed a multistep protocol, beginning with washing the placenta in phosphate-buffered saline (PBS) to remove contaminants. The tissue was then dissected into 1 cm^2^ pieces and subjected to further washing to ensure complete decontamination. The dissected tissue was minced into smaller fragments and divided into two sterile tubes, each undergoing a distinct isolation protocol as described below (Figure 1).

### 2.2. MSC Isolation Protocols

#### 2.2.1. Placental Dissection and Preprocessing

In a Class II safety cabinet, placental tissue was rinsed three times using 20 mL of sterile PBS (Phosphate-Buffered Saline, Gibco, Grand Island, NY, USA) per rinse to remove blood and contaminants. Each washing step lasted 5 min with gentle shaking. The placenta was dissected into 1 cm^2^ pieces using sterile surgical scissors.

A total of 5 g of tissue per placenta was processed, divided equally between two sterile 50 mL Falcon tubes for enzymatic digestion under two different protocols.

#### 2.2.2. Trypsin with Collagenase Protocol

Placental tissue fragments were incubated in 0.025% Trypsin-EDTA solution (Gibco, Grand Island, NY, USA) for 2 h at 37 °C, followed by a second digestion step using Collagenase Type I (Sigma-Aldrich, Burlington, MA, USA) at a concentration of 0.1% for another 2 h at 37 °C. After enzymatic digestion, the tissue was filtered through a 70 µm sterile mesh filter to remove undigested debris. The resulting cell suspension was washed and cultured in Mesenchymal Stem Cell Growth Medium consisting of low-glucose DMEM (Gibco, Grand Island, NY, USA), 10% fetal bovine serum (FBS) (Gibco, Grand Island, NY, USA), 1% Penicillin-Streptomycin (Sigma-Aldrich, Burlington, MA, USA), and 2 mM L-Glutamine (Gibco, Grand Island, NY, USA). The medium was supplemented with basic fibroblast growth factor (bFGF, 5 ng/mL). The cell suspension underwent additional filtration and straining using a 40 µm cell strainer (Corning, Corning, NY, USA) to isolate viable mononuclear cells and remove residual tissue particles.

#### 2.2.3. Trypsin-Only Protocol

Placental tissue was incubated with 0.025% Trypsin-EDTA for 3 h at 37 °C. The digested material was filtered through a 70 µm mesh, then further processed using a 40 µm cell strainer to purify mononuclear cell fractions. The filtered cells were cultured in the same MSC growth medium described above. Forty-eight hours after seeding, MSCs adhered to the bottom of the culture flask. Non-adherent cells and debris were removed by carefully aspirating the medium, followed by three gentle washes with PBS. Fresh complete culture medium was then added to continue cell expansion.

### 2.3. Subculture and Expansion of MSCs

For 2D expansion, MSCs were cultured until reaching 80–90% confluence at a density of 5 × 10^4^ cells/cm^2^ in T-75 flasks containing 15 mL of MSC growth medium. The culture medium was aspirated, and cells were washed with PBS before treatment with 0.025% Trypsin-EDTA for 5 min at 37 °C. Detached cells were neutralized with medium, centrifuged at 300× *g* for 5 min, and resuspended in fresh MSC medium. Cell viability and counts were measured using both a Countess II Automated Cell Counter (Thermo Fisher Scientific, Waltham, MA, USA) and a Thoma-Lam chamber (Marienfeld, Harsewinkel, Germany) with 0.04% Trypan Blue.

### 2.4. Characterization of MSC

Characterization was performed every 5–7 days, depending on the growth rate, and cells were characterized at passage 3. MSCs were analyzed for surface markers by flow cytometry. For surface marker analysis, 1 × 10^6^ cells were incubated with monoclonal antibodies against CD90, CD105, and CD73 (positive markers) and CD14, CD34, CD45, and HLA-DR (negative markers). Fluorescence was measured using a CytoFlex cytometer (Beckman Coulter, Brea, CA, USA) with CytExpert 2.4 software (Beckman Coulter, Brea, CA, USA). These markers align with the International Society for Cellular Therapy (ISCT) criteria for defining human MSCs. CD73, CD90, and CD105 are adhesion and differentiation-related proteins commonly expressed on MSCs, while CD14, CD34, CD45, and HLA-DR are hematopoietic or immune markers used to exclude non-MSC populations.

### 2.5. Two-Dimensional and Three-Dimensional Chondrogenic Differentiation of Mesenchymal Stem Cells

Chondrogenic differentiation was induced using the Mesencult™ Chondrogenic Differentiation Kit (StemCell Technologies, Vancouver, BC, Canada) with additional supplements of 1 mL L-Glutamine and 1 mL Penicillin-Streptomycin.

#### 2.5.1. Two-Dimensional Culture

MSCs were plated at a density of 2 × 10^6^ cells in a T-75 flask in 15 mL of differentiation medium, and the cells were cultured under standard conditions (37 °C, 5% CO_2_). The medium was replaced every 72 h, and differentiation was allowed to proceed for 30 days.

#### 2.5.2. Three-Dimensional Culture

For the 3D differentiation protocol, MSCs were plated at a density of 5 × 10^5^ cells in 15 mL of medium in a 15 mL conical tube. The tube was centrifuged at 350× *g* for 5 min to form a cell pellet. The pellet was cultured under the same conditions as the 2D cultures, with medium changes every 72 h. The culture was maintained for 21 days. After the differentiation period, cultures were assessed under a microscope, and pellets were fixed in paraffin and stained with Alcian Blue for the detection of proteoglycan deposition and glycosaminoglycan.

### 2.6. Three-Dimensional Chondrocyte Cultures Using PLGA Scaffolds

#### 2.6.1. Preparation of PLGA Sheets

PLGA scaffolds were prepared using a solvent casting and particulate leaching method, adapted from established protocols to create porous, biocompatible sheets. Poly(lactic-*co*-glycolic acid) (PLGA, 50:50, inherent viscosity 0.55–0.75 dL/g, Sigma-Aldrich, Burlington, MA, USA) was dissolved in chloroform (10% *w*/*v*) under constant stirring. Sodium chloride (NaCl) particles (250–500 µm) were added as a porogen to achieve desired porosity. The PLGA-NaCl mixture was cast into a flat mold (Teflon-coated Petri dish) and allowed to air-dry in a fume hood for 48 h to evaporate the solvent. The scaffold was dried in a laminar flow hood under sterile conditions at room temperature. The dried PLGA-NaCl sheets were then immersed in deionized water for 72 h to leach out the NaCl and generate porous structures, with water changed every 12 h.

After leaching, sheets were dried at room temperature and cut into 1 × 1 cm^2^ pieces for subsequent cell seeding. Prior to cell culture, the scaffolds were sterilized by soaking in 70% ethanol for 30 min and exposing to UV light for 1 h per side in a laminar flow hood.

The same protocols and culture conditions were used for both the cell-seeded scaffolds and the cell-free controls.

#### 2.6.2. Static 3D Cell Culture of Chondrocytes

**Figure 2:**
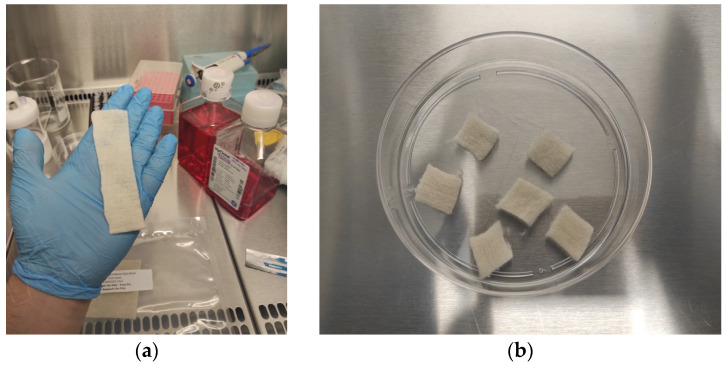
(**a**) PLGA sheet before cutting into 1 cm^2^ pieces. (**b**) PLGA biopolymer pieces cut to 1 cm^2^.

**Figure 3:**
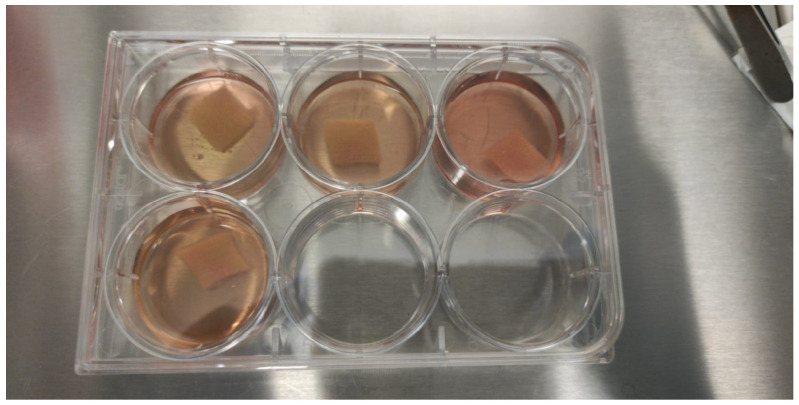
Static 3D culture setup using PLGA biopolymer in a 6-well plate seeded with chondrocytes.

Chondrocytes were seeded onto 1 × 1 cm pieces of PLGA (poly(lactic-*co*-glycolic acid)) scaffold at a density of 1.2 × 10^6^ cells per piece (Figure 2). The seeded PLGA sheets were placed in 6-well plates, each containing 5 mL of DMEM High-Glucose medium supplemented with 10% FBS, 1% Penicillin-Streptomycin, and 10 ng/mL TGF-β1 (Figure 3). The cultures were incubated at 37 °C in a 5% CO_2_ atmosphere for 21 days. Medium changes were performed every 72 h. After the culture period, the PLGA sheets were stained with Hematoxylin-Eosin (H&E) and Toluidine Blue to evaluate cell morphology and ECM production. Staining interpretation was guided by comparison with unseeded control scaffolds to distinguish ECM production from background PLGA absorption.

#### 2.6.3. Dynamic 3D Cell Culture of Chondrocytes

**Figure 4:**
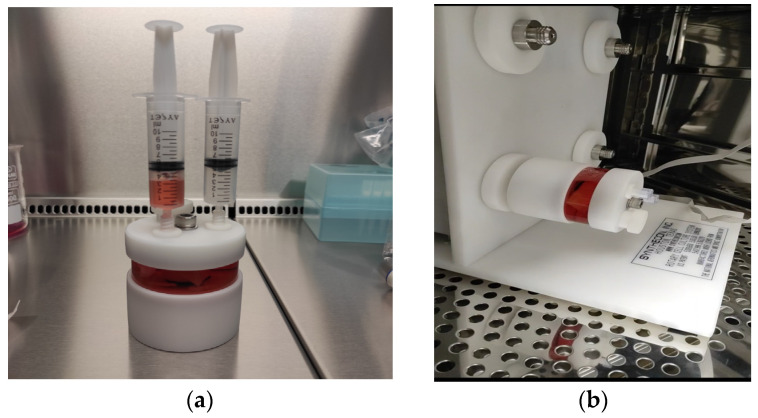
(**a**) Chondrocytes are seeded onto the cut biopolymers, which are then placed in a 55 mL STLV. (**b**) The 55 mL STLV is cultured by integrating it into the RCCS system in the incubator.

For dynamic 3D culture, chondrocytes were seeded onto 1 × 1 cm pieces of PLGA at a density of 1.2 × 10^6^ cells per piece. The seeded pieces were transferred to a Slow-Turning Lateral Vessel (STLV) with a 55 mL capacity, integrated into an RCCS-4HD bioreactor system (Synthecon, Houston, TX, USA). The STLV was filled with 7.5 mL of DMEM High-Glucose medium supplemented with 10% FBS, 1% Penicillin-Streptomycin, and 10 ng/mL TGF-β1. The bioreactor system was maintained at 37 °C with 5% CO_2_, and the STLV was rotated at a speed of 15 rpm to facilitate nutrient diffusion and ECM synthesis (Figure 4). The culture medium was changed every 72 h, and the culture process was continued for 21 days. After incubation, the PLGA scaffolds were harvested, and the samples were stained with Hematoxylin and Eosin (H&E) and Toluidine Blue to assess ECM production and chondrocyte differentiation.

#### 2.6.4. Three-Dimensional Cell Culture for PLGA Degradation

For the 3D static culture, PLGA pieces were incubated in different media (1st well: DMEM High-Glucose medium supplemented with 10% FBS, 1% Penicillin-Streptomycin, and 10 ng/mL TGF-β1, 2nd well: PBS, 3rd well: DMEM Low-Glucose medium supplemented with 10% FBS and 1% Penicillin-Streptomycin, 4th well: DMEM Low-Glucose medium) in a 6-well plate at 37 °C and 5% CO_2_.

For the 3D dynamic culture, PLGA was transferred to a Slow-Turning Lateral Vessel (STLV) with a 55 mL capacity, integrated into an RCCS-4HD bioreactor system (Synthecon, Houston, TX, USA). The STLV was filled with 7.5 mL of PBS. The bioreactor system was maintained at 37 °C with 5% CO_2_, and the STLV was rotated at a speed of 15 rpm.

## 3. Results

### 3.1. Successful MSC Isolation and Attachment

**Figure 5:**
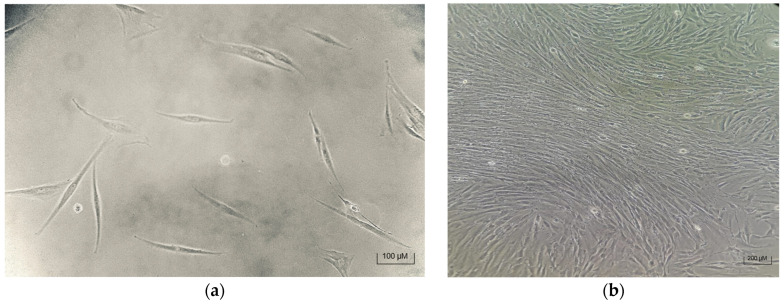
(**a**) MSCs after 48 h of isolation and washing. (**b**) Microscopic image of confluent stem cells ready for passage.

Mesenchymal stem cells (MSCs) were successfully attached to culture flasks within 48 h of plating, exhibiting the typical fibroblast-like morphology characteristic of MSCs (Figure 5). No residual red blood cells or significant debris were observed, indicating effective isolation. Both the Trypsin + Collagenase and Trypsin-only protocols isolated MSCs; however, the Trypsin + Collagenase method yielded a significantly higher cell count, consistent with enhanced enzymatic digestion facilitating better tissue dissociation.

### 3.2. MSC Characterization—Marker Expression Analysis

**Figure 6:**
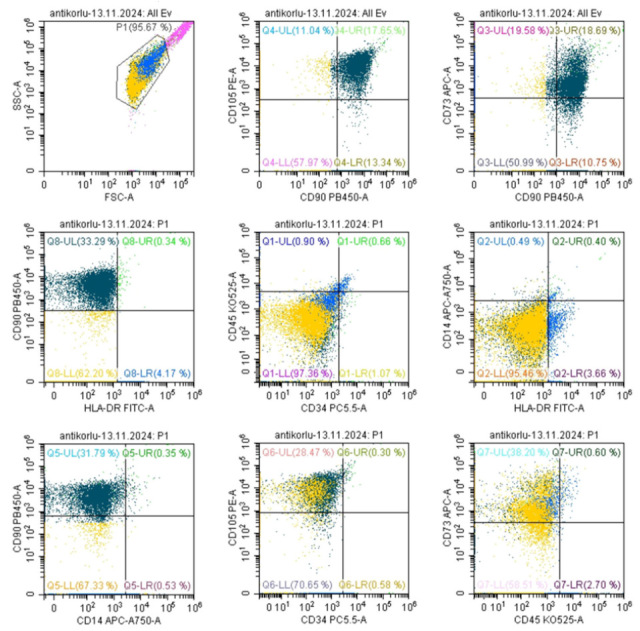
Flow Cytometry Analysis of MSCs at Passage 3: positive for CD73, CD90, and CD105 and negative for CD14, CD34, CD45, and HLA-DR expression.

Flow cytometry of third-passage MSCs confirmed the expected mesenchymal phenotype, with high expression of CD73, CD90, and CD105 and negligible expression of hematopoietic markers CD14, CD34, CD45, and HLA-DR (Figure 6). These markers confirm the purity of the MSC population and their mesenchymal lineage, critical for ensuring the correct cellular phenotype for chondrogenic differentiation.

### 3.3. Successful Chondrocyte Differentiation in 2D and 3D Cultures

**Figure 7:**
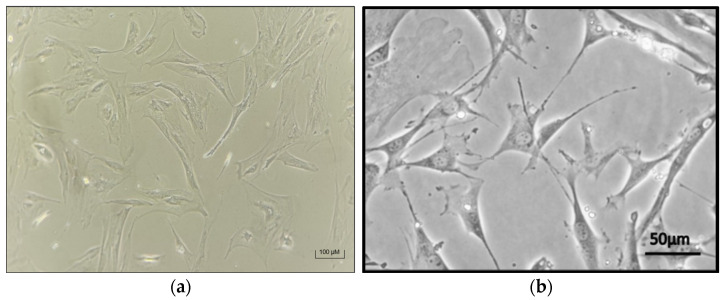
The chondrocytes differentiated from extra-embryonic MSCs in 2D cell culture. (**a**) Image of chondrocytes differentiated from extra-embryonic MSCs in our study. (**b**) Image of chondrocytes differentiated from MSCs in another study [41].

In the 2D monolayer culture system, MSCs underwent successful differentiation into chondrocytes over a 30-day period. Microscopic examination of the culture flasks revealed a loss of the typical fibroblast-like morphology of the MSCs, with cells taking on a rounder appearance, indicative of chondrocyte differentiation. This morphological shift was consistent with chondrogenic differentiation observed in other studies [41] (Figure 7).

**Figure 8:**
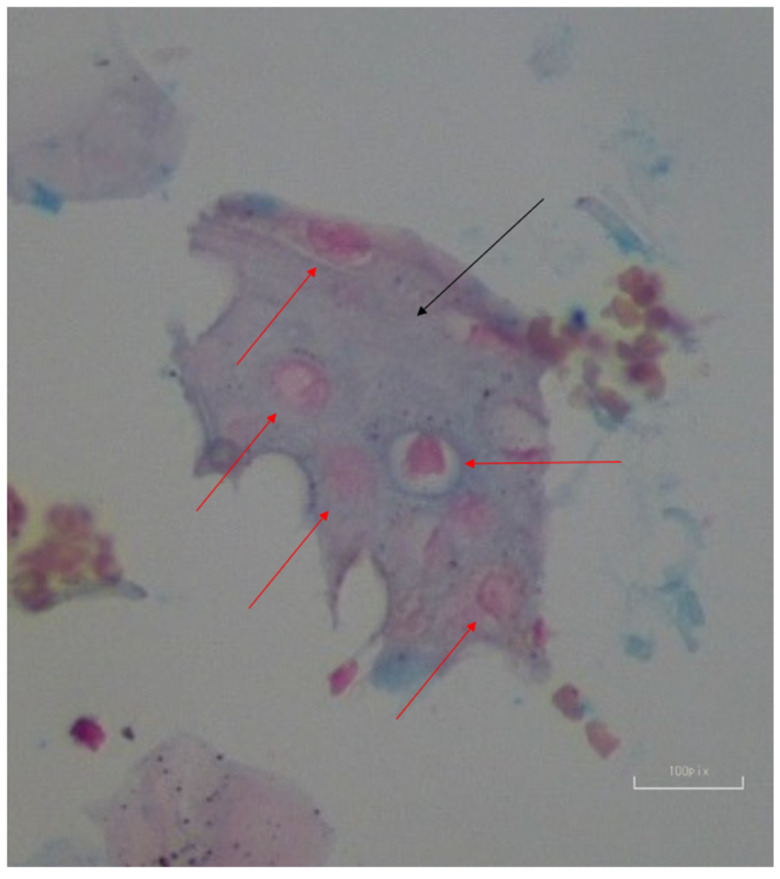
The image resulting from 3-dimensional chondrogenic differentiation, obtained by staining the pellet with Alcian Blue. The red arrows indicate chondrocytes, while the black arrow points to the GAG structure.

In 3D pellet cultures, Alcian Blue staining after 21 days revealed glycosaminoglycan (GAG) deposition, indicating the presence of cartilage-like extracellular matrix (ECM) (Figure 8). To confirm that the Alcian Blue staining corresponds to ECM components and not scaffold artifacts, unseeded PLGA controls stained under the same conditions showed distinct patterns and no GAG-specific staining, supporting the specificity of the staining for chondrogenic ECM.

### 3.4. PLGA Biopolymer Degradation Timeline

**Figure 9:**
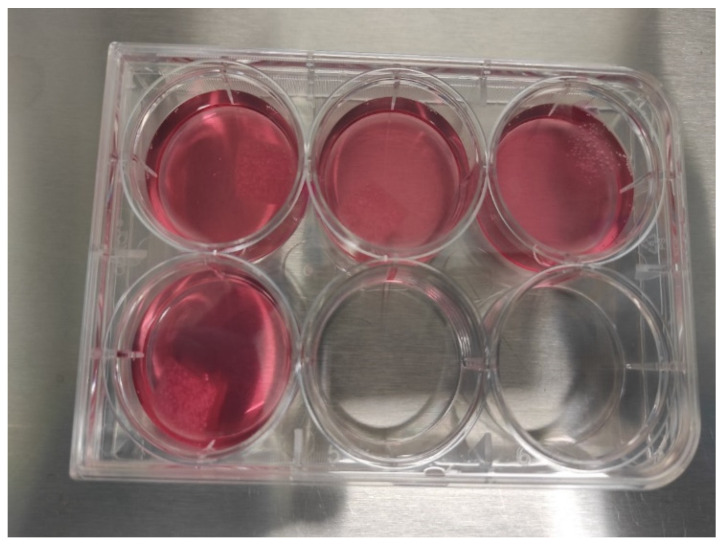
PLGA degraded in 6-well plate on day 6 of 3D static culture.

**Figure 10:**
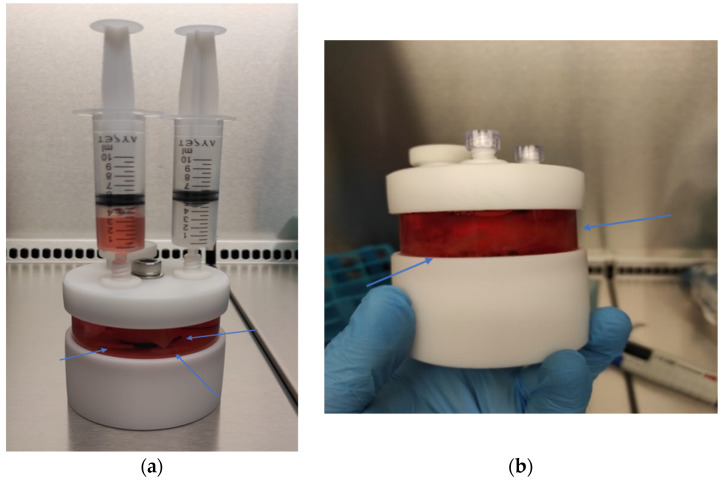
(**a**) Photograph taken during the medium change at hour 72 of D culture. The square-shaped structures at the ends of the blue arrows are non-degraded PLGAs. (**b**) Photograph of PLGA degraded in 55 mL STLV on day 6 of 3D dynamic culture. The structures at the tip of the blue arrow indicate degraded PLGA.

PLGA scaffolds in both static and dynamic 3D cultures began degrading by day 6 and were largely degraded by day 9 (Figure 9 and Figure 10). Notably, PLGA pieces cultured in PBS alone showed no degradation over highlighting the role of cellular activity and media components in polymer breakdown.

The Alcian Blue and Toluidine Blue staining predominantly target sulfated GAGs and cartilage-specific ECM molecules. Control experiments with unseeded PLGA scaffolds demonstrated that the dye uptake by PLGA itself was minimal and produced distinguishable staining patterns, allowing confident interpretation of ECM presence in cell-seeded samples. While ECM components were detected as early as day 6, the majority of ECM deposition and scaffold remodeling occurred over the subsequent days, with optimal ECM maturity expected closer to day 21.

### 3.5. RCCS-Mediated PLGA Biopolymer Degradation

Similar degradation kinetics were observed in the RCCS system, with PLGA degradation commencing by day 6 and being completed by day 9 (Figure 10). The dynamic culture environment may accelerate degradation through enhanced nutrient diffusion and mechanical forces. Despite scaffold degradation, cells largely remained within 3D aggregates or attached to residual scaffold fragments, maintaining a 3D microenvironment.

### 3.6. Assessing ECM Formation in Chondrogenic Tissue

**Figure 11:**
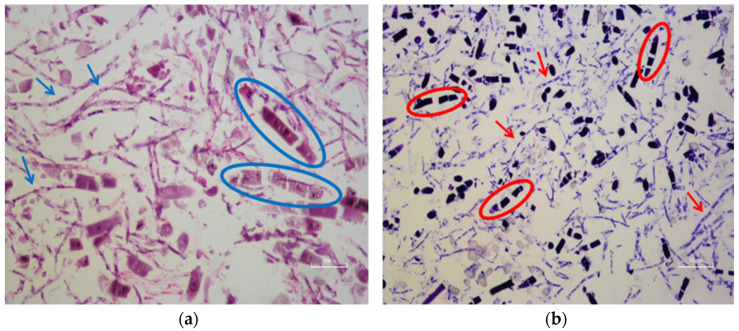
After staining with H&E (**a**) and toluidine blue (**b**), samples obtained from the RCCS on the 9th day of culture displayed normal cartilage tissue morphology. Chondrocytes forming an isogenous group (areas marked as rings) and collagen fibers (indicated by arrows) from the fibrous elements of the cartilage extracellular matrix were observed. Scale bar: 100 pix.

Hematoxylin and Eosin (H&E) and Toluidine Blue staining of samples after 9 days in RCCS culture revealed isogenous groups of chondrocytes surrounded by ECM, including visible collagen fibers, which are consistent with cartilage tissue morphology (Figure 11). The staining patterns were carefully distinguished from PLGA residues by comparison to control scaffolds without cells, where minimal nonspecific dye absorption was observed. Chondrogenic differentiation was primarily confirmed through morphological changes and ECM-specific staining. Also collagen type II as a cartilage-specific marker was used to validate differentiation.

### 3.7. Biopolymer Degradation in 3D Cell Cultures

**Figure 12:**
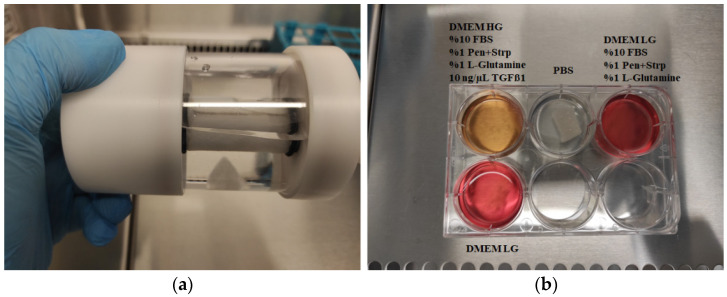
(**a**) Photograph of a PLGA piece cultured with PBS in the RCCS-STLV bioreactor. (**b**) Photograph of PLGA pieces cultured with different culture media.

Segmented PLGA pieces cultured in 6-well plates exhibited degradation by day 6 and complete degradation by day 9 in media containing cells, while no degradation occurred in PBS-only controls (Figure 12). These results confirm that cellular metabolism and media components contribute to PLGA degradation.

The PLGA pieces cultured in the RCCS-STLV bioreactor with PBS alone did not degrade over 21 days, contrasting with static culture observations and suggesting that mechanical conditions and medium composition both influence degradation rates.

## 4. Discussion

The placenta is a valuable and accessible source of mesenchymal stem cells (MSCs), offering numerous advantages for clinical and therapeutic applications. As a non-controversial source of MSCs, derived from postpartum tissue with no ethical concerns, the placenta is a promising alternative to traditional sources like bone marrow or adipose tissue [42,43,44,45,46]. In this study, we successfully isolated MSCs from placental tissue using two different protocols, with the Trypsin + Collagenase protocol proving more efficient in terms of yield. This aligns with previous reports that collagenase, when used in combination with Trypsin, enhances cell isolation by breaking down tissue matrices, thereby improving cell recovery [47].

The characterization of the isolated MSCs revealed typical surface protein expressions consistent with the mesenchymal lineage, including markers such as CD73, CD90, and CD105. These findings are in agreement with the established MSC phenotype described in the literature [45,48,49,50]. Notably, telomerase activity, which is often used as an indicator of the proliferative capacity of MSCs, was not assessed in this study due to the nature of the starting material. However, previous studies have demonstrated that placenta-derived MSCs generally exhibit robust proliferative capabilities and may possess longer telomere lengths compared to MSCs derived from other sources [51,52].

Our study also confirmed the successful chondrogenic differentiation of MSCs into chondrocytes in both 2D and 3D culture systems. The morphological changes observed in the differentiated cells, including the loss of the fibroblast-like shape and acquisition of a rounder chondrocyte morphology, were consistent with findings in other studies [42,53,54]. These results suggest that placental MSCs, like those derived from other tissues, are capable of differentiating into chondrocytes under appropriate culture conditions, demonstrating their potential for cartilage tissue engineering applications.

An unexpected finding in our study was the degradation of the PLGA biopolymer in both static and dynamic 3D culture systems. This phenomenon may have been influenced by various factors, including the composition of the biopolymer, the sterilization methods used, and the local environmental conditions within the cultures [55,56,57]. The degradation of PLGA in tissue engineering scaffolds is known to occur over time as the material undergoes hydrolysis and enzymatic breakdown, which is typically accelerated by the presence of cells secreting various proteases. However, the relatively rapid degradation observed in our experiments warrants further investigation into the specific interactions between the MSCs and the biopolymer and whether this could affect the structural integrity of the final tissue construct.

MSC differentiation is known to be influenced by multiple factors, including the regulation of growth factors and interactions with the surrounding microenvironment. In our study, we focused on TGF-β1, a key growth factor in chondrogenesis. Numerous studies have confirmed that TGF-β1 plays a pivotal role in the differentiation of MSCs into chondrocytes, stimulating the expression of cartilage-specific markers such as collagen II and aggrecan [58,59,60]. Despite the observed biopolymer degradation, we found that TGF-β1 significantly contributed to cartilage formation, as evidenced by the formation of isogenous groups and collagen fibers within the extracellular matrix (ECM). This suggests that TGF-β1 not only induced differentiation but also facilitated ECM synthesis and deposition, which are critical components of cartilage tissue.

The use of bioreactors in tissue engineering has become increasingly important for supporting the growth, differentiation, and maturation of engineered tissues. Compared to traditional static cultures, bioreactors offer several advantages, such as enhanced nutrient and oxygen delivery, better control of mechanical forces, and the ability to sustain larger tissue constructs. Rotating wall vessels (RWVs), for example, provide a unique advantage by inducing fluid flow, mimicking physiological conditions such as shear stress, and offering a model of microgravity. These features promote cellular differentiation and ECM production in 3D cultures, helping to create more robust and functional tissue constructs. In our study, the dynamic culture system in the RCCS-4HD bioreactor played a key role in supporting chondrocyte phenotypic expression and ECM formation, despite the challenges posed by PLGA degradation. The 3D aggregation of cellular elements was confirmed through both histological and confocal analyses. Histological cross-sections, stained with H&E and Safranin O, revealed multilayered cell structures embedded in a dense extracellular matrix, while confocal microscopy imaging further demonstrated spatial organization typical of three-dimensional tissue constructs. These findings collectively validate the successful formation of 3D cartilage-like tissue and support the phenotypic maturation of differentiated chondrocytes within the scaffold. This further emphasizes the utility of bioreactors in advancing tissue engineering, as they facilitate the creation of more physiologically relevant tissues.

Despite the biopolymer degradation, our study underscores the significant contribution of bioreactors in tissue engineering, particularly in facilitating extracellular matrix formation and chondrocyte phenotypic expression, ultimately resulting in cartilage tissue formation [40,61]. This highlights the importance of bioreactors in advancing tissue engineering research

## 5. Conclusions

The utilization of placenta-derived mesenchymal stem cells (MSCs) presents a promising and ethically sound approach in the fields of regenerative medicine and tissue engineering. As a readily accessible and non-controversial source of stem cells, the placenta offers significant advantages over traditional stem cell sources, such as bone marrow or adipose tissue. In this study, we successfully isolated MSCs from placental tissue using two different protocols, with the Collagenase combined with Trypsin protocol demonstrating higher efficiency in terms of cell yield. These findings align with previous studies and further validate the viability of using placental-derived MSCs in therapeutic applications. Characterization of these isolated MSCs revealed typical surface marker expressions (CD73, CD90, CD105) and the absence of markers associated with hematopoietic lineages (CD14, CD34, CD45), confirming their mesenchymal origin and suitability for tissue engineering purposes.

Our investigation into chondrogenic differentiation, in both conventional 2D culture systems and more advanced 3D systems, yielded encouraging results. In both culture conditions, MSCs successfully differentiated into chondrocytes with morphology resembling native cartilage tissue. This suggests the potential of placenta-derived MSCs for cartilage regeneration, a key therapeutic area in orthopedics and regenerative medicine. Notably, the 3D culture system demonstrated enhanced cell aggregation, ECM production, and phenotypic expression of chondrocytes, further highlighting the advantages of 3D culture systems in recapitulating the complex in vivo tissue environment.

However, an unexpected challenge arose with the degradation of PLGA biopolymer scaffolds during both static and dynamic 3D cultures. While the degradation of these biopolymer scaffolds is a well-known phenomenon, it was noteworthy in our study that degradation occurred earlier than anticipated. This observation underscores the need for further investigation into the influence of scaffold composition, sterilization methods, and environmental factors on the longevity and stability of biopolymer scaffolds in 3D tissue cultures. Despite this challenge, our findings also emphasize the crucial role of bioreactor systems, particularly the use of the Slow-Turning Lateral Vessel (STLV) in the RCCS-4HD bioreactor, in facilitating optimal conditions for chondrocyte growth and extracellular matrix (ECM) formation. Bioreactors, with their ability to mimic dynamic in vivo conditions, support nutrient diffusion, and enable the regulation of oxygen and shear forces, are pivotal in advancing tissue engineering by creating functional tissue constructs that better resemble natural tissues.

In conclusion, our study highlights the potential of placenta-derived MSCs as a valuable cell source for regenerative medicine applications, especially in cartilage tissue engineering. The combination of these MSCs with 3D culture systems and bioreactor technology holds great promise for improving the efficacy of tissue engineering strategies. To fulfil the potential of this approach, future studies should focus on optimizing biopolymer compositions, exploring various growth factor protocols, and refining bioreactor parameters to enhance tissue development. As this field advances, it could lead to innovative therapeutic interventions for cartilage repair, offering hope for patients suffering from degenerative joint diseases and traumatic cartilage injuries.

## Data Availability

The original contributions presented in this study are included in the article; further inquiries can be directed to the corresponding author/s.

## Abbreviations

The following abbreviations are used in this manuscript:

MSC	Mesenchymal Stem Cell
2D	2-Dimensional
3D	3-Dimensional
PGA	Poly(glycolic acid)
PLA	Poly(lactic acid)
PLGA	Poly(lactic-*co*-glycolic acid)
PCL	Polycaprolactone
RCCS	Rotary Cell Culture Systems
ECM	Extracellular Matrix
STLV	Slow-Turning Lateral Vessel
PBS	Phosphate-Buffered Saline
FBS	Fetal Bovine Serum
H&E	Hematoxylin and Eosin
TGF-β1	Transforming Growth Factor beta-1
GAG	Glycosaminoglycan
RWV	Rotating wall vessel
